# Evaluation of antibiofilm properties of dehydroacetic acid (DHA) grafted spiro-oxindolopyrrolidines synthesized via multicomponent 1,3-dipolar cycloaddition reaction

**DOI:** 10.1038/s41598-023-42528-w

**Published:** 2023-09-15

**Authors:** Adukamparai R. Suresh Babu, Akanksha Sharma, M. P. Athira, Hema K. Alajangi, A. R. Naresh Raj, Janeka Gartia, Gurpal Singh, Ravi Pratap Barnwal

**Affiliations:** 1 Department of Chemistry, Anna University, Chennai, 600025 India; 2 Department of Biophysics, Panjab University, Chandigarh, 160014 India; 3 University Institute of Pharmaceutical Sciences, Panjab University, Chandigarh, 160014 India; 4 Department of Chemistry, IISER, Mohali, Sahibzada Ajit Singh Nagar, Punjab 140306 India; 5 Department of Chemistry, Dwaraka Doss Goverdhan Doss Vaishnav College, Arumbakkam, Chennai, 600106 India; 6 School of Biotechnology, Kalinga Institute of Industrial Technology (KIIT), Bhubaneswar, Odisha 751024 India; 7 Department of Chemistry, University of Calicut, Thenhipalam, 673635 India

**Keywords:** Peptides, Proteins, Biofilms, Molecular modelling

## Abstract

The current work involves the use of dehydroacetic acid based chalcone derivatives for the synthesis of spirooxindole grafted pyrrolidine moieties. All the synthesized compounds have been characterized using spectroscopic techniques such as NMR (^1^H-NMR and ^13^C-NMR), IR, mass and elemental analysis. Molecular mechanics studies were performed to comprehend the regioselectivity in the product formation. Molecular docking of the synthesized compounds was performed with few bacterial proteins of *Bacillus subtilis* and *Pseudomonas aeruginosa* responsible for biofilm formation followed by molecular dynamics simulations with the potential lead compound. Further, to corroborate the results obtained via in silico study, anti-biofilm activity etc. of the synthesized compounds (**4a**–**e**) was checked for effectiveness against biofilm formation. Taken together, this study opens up to explore these compounds’ multiple roles in diverse fields in the arena of medical sciences.

## Introduction

A multicomponent reaction (MCR) involves the combination of three or more components in a reaction to facilitate synthesis of final product(s) exhibiting characteristic features of all the reactants. MCRs are helpful for quick preparation of structurally dissimilar and complex organic compounds in a single reaction^1–10^ and are crucial for diversity-oriented and combinatorial synthesis^11–18^. Thus, MCRs are good alternatives to sequential multi-step synthesis. The initial product derived from MCR serves as a hub for several novel acyclic or cyclic scaffolds via different secondary transformations. In contrast, conventional non-MCR compounds exhibit lower hit rates during screening, for instance, for protein–protein interactions^19^. Among MCRs, the assembly of five-membered heterocyclic rings, particularly spiro-pyrrolidines and pyrrolidines is conveniently carried out with the help of [3 + 2]-cycloaddition of azomethine ylides to olefinic dipolarophiles^20–22^. Spiro scaffolds are in use for various drug discovery efforts due to their structural novelty and three-dimensionality^23,24^. Many spiroxindolopyrrolidines are local anesthetic^25^, anti-inflammatory^26^, HT_1A_/5-HT_2A_ receptor^27^, antileukemic^10^, antiaryhthmic^28^, anti-viral^29^ and anticonvulsant agents^30^ and are also constituents of many biologically active compounds^31^. Various strategies are in use for synthesis of Spirooxindoles^32–36^, considered a promising scaffold for drug discovery^37^. A few drugs containing spirocyclic pyrrolidine are available in the market. Among these are included FDA-approved Spirapril for treating hypertension and Ledipasvir, an inhibitor of non-structural protein of the hepatitis C virus^38^. Moreover, spirocyclic pyrrolidines are also key intermediates in the synthesis of many therapeutic drugs. For instance, spirocyclic pyrrolidine is an intermediate of the antibacterial drug sitafloxacin^39^.

In organic synthesis, dehydroacetic acid (DHA) is a versatile compound used to synthesize various heterocyclic moieties. The IUPAC name of dehydroacetic acid is 3-Acetyl-4-hydroxy-6-methyl-2H-pyran-2-one^40^. Dehydroacetic acid can be utilized for synthesizing several biologically active compounds^41^. The products derived from dehydroacetic acid have multiple applications in the food, cosmetic and pharmaceutical industry^42^. DHA is among the most commonly used acetic acid for preservation purposes. Moreover, it is highly effective against bacteria and yeast and, thus is an excellent antimicrobial agent for various products^43^. Packaging of some cheeses include the addition of sorbic acid or dehydroacetic acid as preservatives^44^. 1,2,3-triazole-linked dehydroacetic acid-chalcone hybrids have previously been synthesized and examined for their antibacterial and antifungal activity. Many of these compounds exhibited better activity than the drugs already in use against the pathogens tested^45^. Chalcones have been used as chemoprotective agents due to their antioxidant properties. Moreover, chalcones have also been reported to work as anti-invasive agents besides their ability to inhibit Nitric Oxide (NO), which is an effector of cytotoxicity induced by macrophages^46^. DHA has also been used as a food additive and as a stabilizer for various cosmetic products. DHA is also widely used as a herbicide and antimicrobial against yeasts, bacteria, molds etc.^47,48^. DHA-based chalcones exhibit antitumor, antifungal, anticancer, anti-inflammatory, anti-oxidant, immune-suppression, cytotoxicity, antibacterial and antiprotozoal activities. Besides, DHA is also used for its anti-viral, antimalarial, and anti-hyperglycemic activities^49–51^. Due to the high chemical reactivity and good physiological properties of dehydroacetic acid, it has applications in many fields.

A comparison in structures of the new synthesized compounds **4a**–**e** with the previously synthesized spiro-oxindolopyrrolidines^52^ reveals that both the series have spiro-oxindolopyrrolidine framework, but the difference lies in the substituent at the C-3 position of the spiro-pyrrolidine ring. In one case, the C-3 position has spiro-oxindole, while in the current work, the C-3 position of the pyrrolidine has pyranone ring as one of the substituent. Spiro-oxindole at C-2 position of the pyrrolidine ring showed hydrogen bonding with the amino acid residue of S12 subunit of the ribosomal protein. The Spiro-oxindole at C-2 position of the pyrrolidine ring in the compounds **4a**–**e** showed hydrogen bonding with residues of TasA and TapA proteins responsible for biofilm formation. It was interesting to note that in both the series of compounds even though the core structure of the compounds is the same (spiro-oxindolopyrrolidines), the degree of interaction and their binding energies were found to be different.

The potential of these heterocyclic entities has encouraged us to synthesize hybrid heterocyclic systems incorporating dehydroacetic acid, oxindole and pyrrolidine in a single molecule. The syntheses of the products were accomplished in one pot without using catalyst or any specific reaction conditions in order to improve the yield of the products unlike some other complex multicomponent component reactions which we have previously carried out^53–57^. The yields of the products were also satisfactory without employing such strategy. In order to check antibacterial activity of the synthesized compounds **4a**–**e** against bacterial pathogens via in silico approaches, a few proteins of bacterial pathogens *Bacillus subtilis* and *Pseudomonas aeruginosa* have been selected for performing molecular docking studies and molecular dynamics (MD) simulations. Further, in vitro studies including minimum inhibitory concentration (MIC) assay to check minimum inhibitory concentrations of synthesized compounds against *B. subtilis* and *P. aeruginosa* as well as biofilm assay to check their antibiofilm properties against these microorganisms are being reported for the first time. *Bacillus subtilis* is a Gram-positive bacterium extensively involved in biofilm formation, which serves to protect the bacteria against unfavorable conditions. *Pseudomonas aeruginosa* is a Gram-negative bacterium, ubiquitously present in the environment. It is multi-drug resistant and known to cause nosocomial infections in humans^58^. Additionally, hemolytic assay and cell viability assay are also done for the first time to determine the toxicity of the synthesized compounds on human erythrocytes and in cell culture, which highlights the potential of these compounds to be tested in vivo. We show here a list of hybrid heterocyclic systems incorporating dehydroacetic acid, oxindole and pyrrolidine. All the synthesized compounds (**4a**–**e**) exhibited promising antibacterial activity besides minimum toxicity to human RBCs and cell lines. This also opens to explore their multiple roles in diverse fields which are currently under investigations in our lab.

## Results and discussion

### Synthesis of dehydroacetic acid grafted spiro-oxindolopyrrolidine heterocycles 4a–e

**Figure 1:**
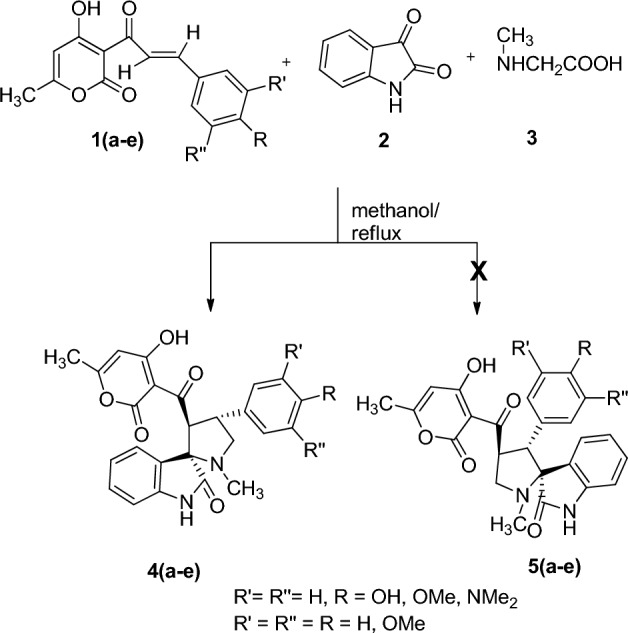
Representation of One-pot synthesis of highly substituted spiroheterocyles containing oxindole and pyrrolidine moieties using dehydroacetic acid based chalcone derivatives 1 (a–e), isatin 2 and sarcosine 3.

The current work reports one-pot synthesis of highly substituted spiroheterocyles containing oxindole and pyrrolidine moieties using various unusual dehydroacetic acid-based chalcone derivatives **1(a**–**e)**, isatin **2** and sarcosine **3** (Fig. 1). This is a continuation of our group’s research in cycloaddition reactions^52,56,57,59–62^.

**Figure 2:**
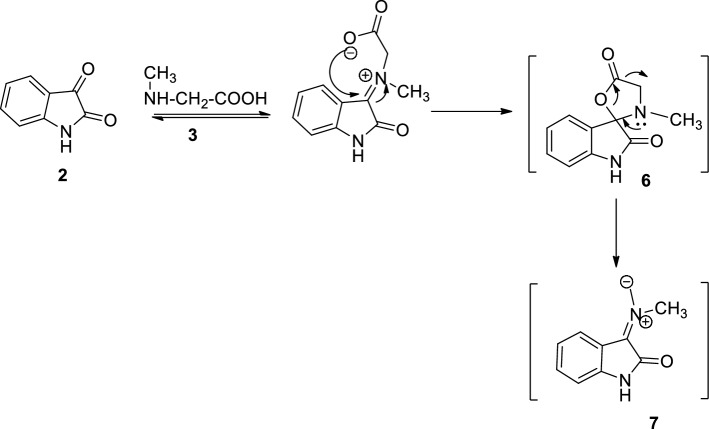
Representation of the synthesis of 1,3-dipole, azomethine ylide **7** involving reaction of isatin **2** with sarcosine **3** via thermal decarboxylation of **6**.

The one-pot three-component reaction involving dipolarophile **1a**, isatin **2** and sarcosine **3,** was performed in methanol at reflux temperature to produce dehydroacetic acid grafted spiro-oxindolo pyrrolidine **4a**. Isatin **2** reacted with sarcosine **3** to give 1,3-dipole, azomethine ylide **7** via thermal decarboxylation of **6**^56,62,63^. The azomethine ylide thus formed is shown in Fig. 2. Non-stabilised azomethine ylide synthesis yields iminium carboxylate betaine **6** followed by decarboxylation.

Cycloaddition of azomethine ylide **7** across the exocyclic double bond of the dipolarophile **1a** results in 4a in good yield (Fig. 1). The IR spectrum of the cycloadduct **4a** exhibited peak at 1686 and 1710 cm^−1^ due to the carbonyl group and at 3462 cm^−1^ due to the –NH group of the oxindole ring (Supplementary Fig. 11). The UV–Visible spectra of cycloadduct **4a** revealed a prominent absorption peak at 330 nm. The ^1^H NMR spectrum of product **4a** exhibited a doublet at δ 2.05 (*J* = 0.4 Hz) due to –CH_3_ group of the pyranone ring and a singlet at δ 2.18 due to the pyrrolidine –NCH_3_ protons. The two pyrrolidine –NCH_2_ protons exhibited a doublet of doublet peak at δ 3.32 (*J* = 7.4 Hz, 1.2 Hz) and δ 3.67 (*J* = 8.8 Hz, 2.0 Hz). The methoxy protons give a singlet at δ 3.77. The pyrrolidine ring proton attached to the carbonyl group exhibited a doublet at δ 4.75 (*J* = 8.8 Hz), while pyrrolidine ring proton of the aryl moiety appeared as multiplet in δ 4.47—4.54 ppm.. The vinyl –CH proton of the pyranone ring exhibited a doublet at δ 5.71 (*J* = 0.8 Hz). The –NH proton of the oxindole ring exhibited a singlet at δ 7.54. The aromatic rings protons exhibited doublets at δ 6.74 (*J* = 7.6 Hz), 6.98 (*J* = 7.2 Hz) and multiplet in the region δ 6.68—6.88, 7.09—7.13 and 7.49—7.51 ppm (Supplementary Figs. 12 and 13). The off-resonance proton decoupled ^13^C spectrum of **4a** exhibited peaks at 20.46 and 34.44 ppm due to the CH_3_ group of the pyranone and pyrrolidine ring. The pyrrolidine –NCH_2_ carbon resonated at 42.81 ppm. The pyrroldine ring carbon attached to the aryl moiety and the carbonyl group resonated at 55.28 and 60.03 ppm. The methoxy carbon resonated at 64.37 ppm. The spirocarbon resonated at 73.07 ppm. The carbonyl group of the pyranone ring resonated at 168.94 ppm. The carbonyl group of oxindole resonated at 179.45 whereas the carbonyl group of pyrrolidine ring showed resonance at 204.24 ppm. All other carbons exhibited chemical shifts as per the structure (Supplementary Fig. 14). Mass spectral and elemental analysis confirmed product formation. The LC-mass spectrum (positive ionization mode) of **4a** showed a molecular ion peak at 461.25 [M + H]^+^ (Supplementary Figs. 15 and 16).

For the purpose of optimizing and improving the product yield, the reaction was performed using various other solvents (Table 1). Even during refluxing for a long period in toluene, (10 h), there was no significant increase in product yield (14%). However, refluxing in methanol gave better chemical yield (reaction time: 2.3 h; chemical yield: 86%). Hence, for one-pot MCR with various other dipolarophiles (**1b**–**e**), isatin **2** and sarcosine **3** affording dehydroacetic acid grafted spiro-oxindolopyrrolidines (**4b**–**e**) (Supplementary Figs. 17–38) in good yield, methanol was chosen as a solvent (Table 1).

**Table 1:** Optimization of solvent for the reaction involving dipolarophile **1a**, isatin **2** and sarcosine **3**.

S. no	Solvent^a^	Time (h)	Yield^b^_(%)
1	Toluene^c^	10	14
2	1,4-dioxane	10	63
3	Tetrahydrofuran (THF)	10	34
4	Acetonitrile	5.5	75
5	Methanol	2.3	86

^a^Reaction condition: **1a**, **2** and **3** (1 mmol) in solvent at reflux temperature; ^b^Product yield (in %); ^c^Dean-stark apparatus was used for the reaction.

**Figure 3:**
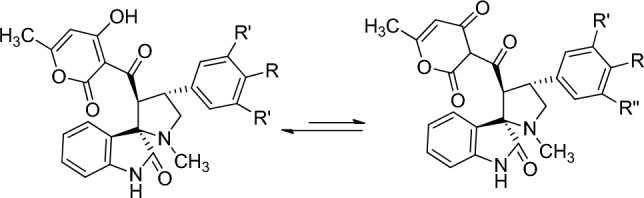
Keto-enol tautomerism in the pyranone ring.

The dehydroacetic acid ring may exist in keto form or enol form. The enol form is the most stable tautomer^64^ as it is well documented in the literature^64,65^ (Fig. 3).

**Figure 4:**
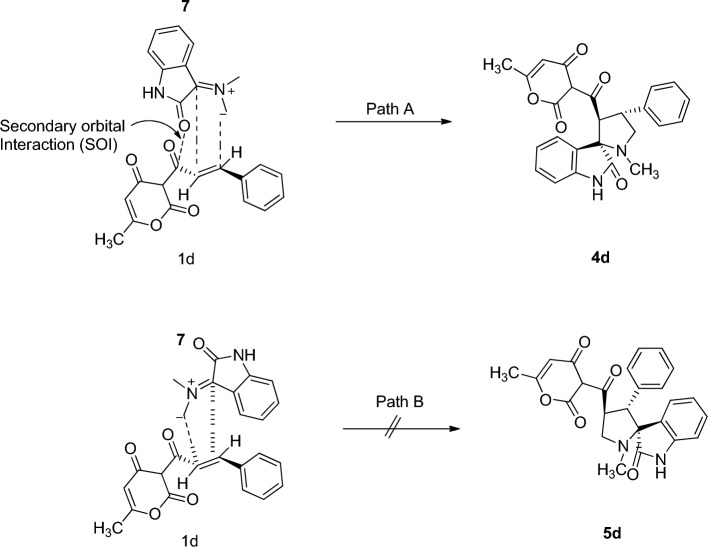
Secondary orbital interaction (**SOI**) approach of azomethine ylide 7.

Thus, the IR spectrum of the cycloadduct **4d** exhibited peak at 1698 and 1715 cm^−1^ due to the carbonyl group and at 3440 cm^−1^ due to the –NH group of the oxindole ring (Supplementary Fig. 28). The UV–Visible spectra of the cycloadduct **4d** revealed a prominent absorption peak at 329 nm. The ^1^H NMR spectrum of the product **4d** exhibited a doublet at δ 2.05 ppm (*J* = 0.8 Hz) due to –CH_3_ group of the pyranone ring and a singlet at δ 2.19 due to the pyrrolidine –NCH_3_ protons. The two pyrrolidine –NCH_2_ protons exhibited a doublet of doublet peak at δ 3.36 (*J* = 7.4 Hz, 1.2 Hz) and δ 3.72 (*J* = 8.8 Hz, 2.0 Hz). The pyranone ring proton attached to the carbonyl group exhibited a singlet at δ 3.49, confirming keto-form of the pyranone ring. The pyrrolidine ring proton attached to the carbonyl group exhibited a doublet at δ 4.81 (*J* = 9.2 Hz), while the one attached to the aryl moiety appeared as multiplet in the region δ 4.52–4.59 ppm. The vinyl –CH proton of the pyranone ring exhibited a doublet at δ 5.71 (*J* = 0.8 Hz). The –NH proton of the oxindole ring exhibited a singlet at δ 7.66 ppm. The aromatic rings protons exhibited a doublet at δ 6.76 (*J* = 7.6 Hz), a triplet at 6.99 (*J* = 3.3 Hz) and multiplet in the region δ 6.84–6.88, 7.09–7.14, 7.19–7.23, 7.29–7.33 and 7.57–7.59 ppm (Supplementary Fig. 29). The off-resonance proton decoupled ^13^C spectrum of **4d** exhibited peaks at 20.46 and 34.44 due to the CH_3_ group of the pyranone and pyrrolidine ring. The pyrrolidne –NCH_2_ carbon resonated at 43.54 and 50.89 ppm. The pyrrolidine ring carbon attached to the aryl moiety and the carbonyl group resonated at 59.94 and 64.18 ppm. The spirocarbon resonated at 73.09 ppm. The two carbonyl group of the pyranone ring resonated at 168.94 and 180.19 ppm. The carbonyl group of oxindole resonated at 179.49, whereas the carbonyl group which is attached to the pyrrolidine ring resonated at 204.10 ppm. All other carbons exhibited chemical shifts in agreement with the proposed structure (Supplementary Fig. 30). Elemental and mass spectral analysis confirmed the product formation. The LC-mass spectrum (positive ionization mode) of **4d** showed a peak at 431.70 [M + H]^+^ (Supplementary Figs. 31 and 32). The regioselectivity can be explained by secondary orbital interaction (**SOI**) of the orbital of dipolarophile **1a** (specifically the carbonyl group) with those of the azomethine ylide **7** (Fig. 4). Accordingly, **4d** via path A is more favorable due to SOI not possible in path B.

### Molecular mechanics

Molecular mechanics force fields are crucial for the study of conformational flexibility and are used for protein simulations. MM potential energy functions allow the precise representation of dispersion interactions and MM force fields also reduce simulations cost by approximating quantum mechanical energy surface with the classical model^66^. MM2 is used to estimate a molecule’s energy as a function of its conformation. The structures with minimized energy are represented in kcal/mol. In the current work, MM2 calculations have been performed for synthesizing **4a**–**e** hybrids^67–70^. Molecular mechanics studies were performed for all the five newly synthesized pyranone grafted spiro-oxindolopyrrolidines giving different values of energy based on the stereochemical aspects and conformation of pyrrolidine ring and substituents on the pyrrolidine ring^70^.

**Figure 5:**
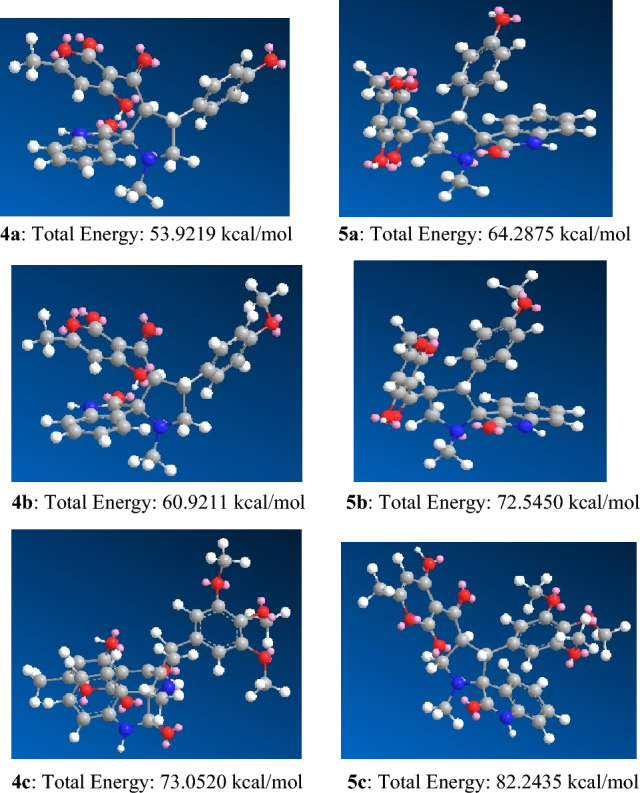
MM2 calculation showing the energy minimized structure and total energy (kcal/mol) for the products **4a**–**c** and their possible regioisomers **5a**–**c** (**Enol form**).

The total energy of the expected products **4a**–**c** ranges from 53 to 73 kcal/mol whereas the total energy of their possible regioisomers **5a**–**c** ranges between 64 and 82 kcal/mol (Fig. 5). A comparison of the total energy of the expected product and its possible regioisomer reveals that the product possesses lesser energy than its possible regioisomer, thus favoring its formation predominantly. It is also significant to note that the total energy of the individual products is different due to different substituents on the hybrid heterocycle.

**Figure 6:**
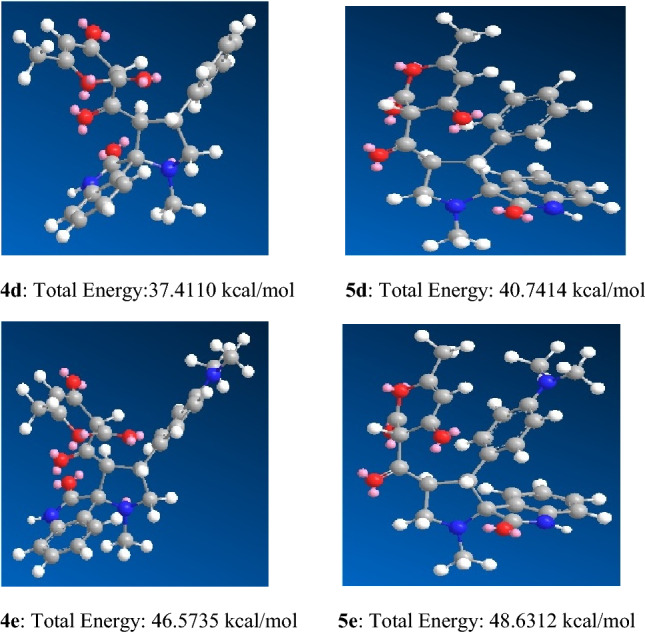
MM2 calculation showing the energy minimized structure and total energy (kcal/mol) for the expected product **4d**–**e** and their possible regioisomers **5d**–**e** (**Keto form of DHA ring**).

From Fig. 6, it is observed that the expected product **4d** and **4e**, where the DHA ring is in keto form as evident by their ^1^H NMR spectrum, has lesser energy than its possible regioisomers **5d** and **5e** thus favoring its formation.

### Molecule docking studies

**Figure 7:**
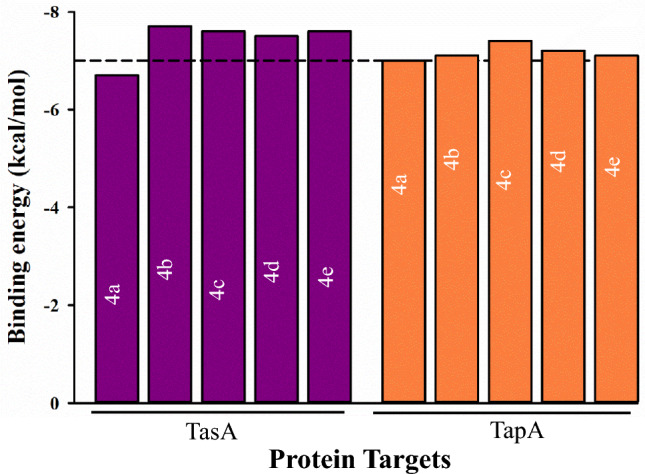
Comparison of binding energy values for docking of the synthesized compounds (4a–e) with the selected protein targets (TasA and TapA). Almost all the compounds exhibited binding energy values above or equal to the reference line, indicating good binding with the target proteins.

Molecular dockings of all the synthesized compounds with *B. subtilis* proteins TasA (PDB: 5OF1) and TapA (PDB: 6HQC) and *P. aeruginosa* protein RetS kinase (PDB: 6DK8) (data shown in Supplementary Figs. 5 and 6) involved in the formation of biofilm have been performed using AutoDock Vina to check the anti-biofilm activity of the compounds. The result files thus generated have been used for analysis by the PLIP webserver. The binding of the macromolecules with the respective ligands have been depicted in figures generated using PyMol. Further, 2D interaction figures are made using LigPlot^+^ software (Source: https://www.ebi.ac.uk/thornton-srv/software/LigPlus/ version: v2.2) to depict hydrophobic interactions and hydrogen bonding (along with hydrogen bond distance)^81^. Molecular Dynamics (MD) studies for the macromolecule-ligand complexes are in progress. The binding energies obtained for protein–ligand dockings (for *B. subtilis* proteins) have been depicted graphically in Fig. 7.

#### Molecular docking and analysis of *Bacillus subtilis* protein TasA with synthesized compounds

Dockings of the TasA protein (PDB: 5OF1) were performed with all the synthesized compounds. The binding energies of 5OF1 with **4a**, **4b**, **4c**, **4d** and **4e** were − 6.7, − 7.7, − 7.6, − 7.5 and − 7.6 kcal/mol, respectively. Various other parameters like inhibition constants for these interactions, amino acids forming hydrogen bonds and hydrophobic interactions were also noted and are summarized in Table 2. The inhibition constant for the interaction of the TasA protein with **4c** was 2.64 µM. All the compounds show good binding with the TasA protein, thus highlighting their potential as antibacterial agents. ASN30 and LEU44 are involved in hydrophobic interactions with the ligand 4c, which shows the highest activity against *B. subtilis* biofilm in vitro. ASN30, ASP31, SER34, THR43 and LYS68 form hydrogen bonds with **4c**. ASN30 formed hydrogen bonds as well as hydrophobic interactions. Interestingly, ASN30 is involved in hydrophobic interactions as well as hydrogen bonding with all the ligands. Similarly, ASP31 forms hydrogen bonds with all the ligands. The three-dimensional figure depicting the binding of these compounds with the target protein have been shown as Supplementary Fig. 1 generated using PyMol. Additionally, H-bonding and hydrophobic interactions of all the compounds (**4a**–**e**) with TasA protein depicted using LigPlot^+^ have been shown in Supplementary Fig. 2.

**Table 2:** Table depicting binding energy values (in kcal/mol), inhibition constants (µM) and various residues of TasA involved in hydrogen bonding and hydrophobic interactions with ligands **4a**–**e**.

Compound	Protein (TasA (PDB: 5OF1))			
	Binding energy (kcal/mol)	Inhibition constant (µM)	Hydrophobic interactions	Hydrogen bonding
**4a**	− 6.7	12.1	ASN30, ASP31, ASP45, LYS49, ALA53, PHE70	ASN30, ASP31, ASP45, LYS49, ASN51
**4b**	− 7.7	2.23	ASN30	ASN30, ASP31, SER34, THR43, GLU50
**4c**	− 7.6	2.64	ASN30, LEU44	ASN30, ASP31, SER34, THR43, LYS68
**4d**	− 7.5	3.13	ASN30	ASN30, ASP31, SER34, THR43, LYS68
**4e**	− 7.6	2.64	ASN30, ALA40, LYS49, GLU50	ASN30, ASP31, SER34, THR43, LYS68

#### Molecular dockings and analysis of *Bacillus subtilis* protein TapA with synthesized compounds

Molecular dockings of the TapA protein (PDB: 6HQC) with the compounds (**4a**–**e**) were performed. **4c** exhibited lowest binding energy (− 7.4 kcal/mol), with inhibition constant value of 3.71 µM. While for both **4b** and **4e**, the binding energy value was − 7.1 kcal/mol. Notably, LYS125, PRO126, LEU127, PHE177, and TRP179 engage in hydrophobic interactions with the compound **4c**. LEU127, LYS166, SER175, TRP179 are involved in hydrogen bonding interactions with **4c**. Remarkably, PRO126 is involved in hydrophobic interactions with all the compounds except **4d**. LEU127 forms hydrogen bonding with all the compounds. Binding energies, inhibition constant values as well as information about residues involved in hydrophobic and H-bond interactions with these compounds are given in Table 3. Macromolecule-ligand binding for TapA with **4a**–**e** visualized using PyMol are shown in Supplementary Fig. 3. Further, 2D interaction plots of all these compounds (**4a**–**e**) with the target protein prepared using LigPlot^+^ are shown in Supplementary Fig. 4.

**Table 3:** Binding energies, inhibition constants and various amino acids of TapA involved in hydrogen bonding and hydrophobic interactions with ligands 4a–e.

Compound	Protein (TapA (PDB: 6HQC))			
	Binding energy (kcal/mol)	Inhibition constant (µM)	Hydrophobic interactions	Hydrogen bonding
**4a**	− 7	7.29	PRO126, PHE177, GLU178	LEU127, SER175, TRP179
**4b**	− 7.1	6.16	LYS125, PRO126, LEU127, PHE177, GLU178, TRP179	LEU127, SER175, TRP179
**4c**	− 7.4	3.71	LYS125, PRO126, LEU127, PHE177, TRP179	LEU127, LYS166, SER175, TRP179
**4d**	− 7.2	5.2	PHE177, TRP179	LEU127, TRP179
**4e**	− 7.1	6.16	LYS125, PRO126, LEU127, PHE177, TRP179	LEU127, SER175, TRP179

The molecular mechanics and molecular docking study performed in the current manuscript showed that irrespective of the energy of individual compounds (Figs. 5, 6), it the efficient binding of the compound with a particular protein giving high negative binding energy value that is crucial for pursuing in vitro or in vivo studies for ascertaining their antibacterial properties. It is interesting to observe that compound **4b** (E = 60.921 kcal/mol) (Fig. 5) showed the best binding affinity with TasA protein, exhibiting binding energy of (E = − 7.7 kcal/mol) and compound **4c** (E = 73.0520 kcal/mol) (Fig. 5) showed the best binding affinity with TapA protein exhibiting binding energy of (E = − 7.4 kcal/mol). High negative binding energy shows effective binding of the compound with the protein which is significant for antibacterial activity (Tables 2, 3). The studies are in accordance with the docking and antibiofilm studies performed by us.

### Molecular Dynamics (MD) simulations for TasA-4c and TapA-4c complex

MD simulations were performed for free TasA protein and free TapA protein as well as for TasA protein with 4c and TapA protein with **4c** at 100 ns. The root mean square deviation (RMSD), root mean square fluctuation (RMSF) and radius of gyration (Rg) are used to analyze the data thus obtained. Both RMSD and RMSF are used to determine structural fluctuations. RMSD refers to the average displacement of atoms with respect to the reference structure at any point of the simulation, typically the first frame of the simulation. RMSD is a measure of average distance between the coordinates and is used to examine whether the structure is stable during the simulations or deviating from the coordinates. Smaller deviation reflects higher stability of the protein structure. RMSD values for a protein typically range from 3 to 4 Å^71^. RMSF measures displacement of a particular atom relative to the reference structure averaged over the total number of atoms^72^. It is used to determine average residue fluctuations during simulation^73^. The radius of gyration (Rg) is used to evaluate flexibility and compactness of the protein in a biological environment. Rg analysis provides information about overall protein dimensions^74^.

#### MD simulation of TasA-4c complex

**Figure 8:**
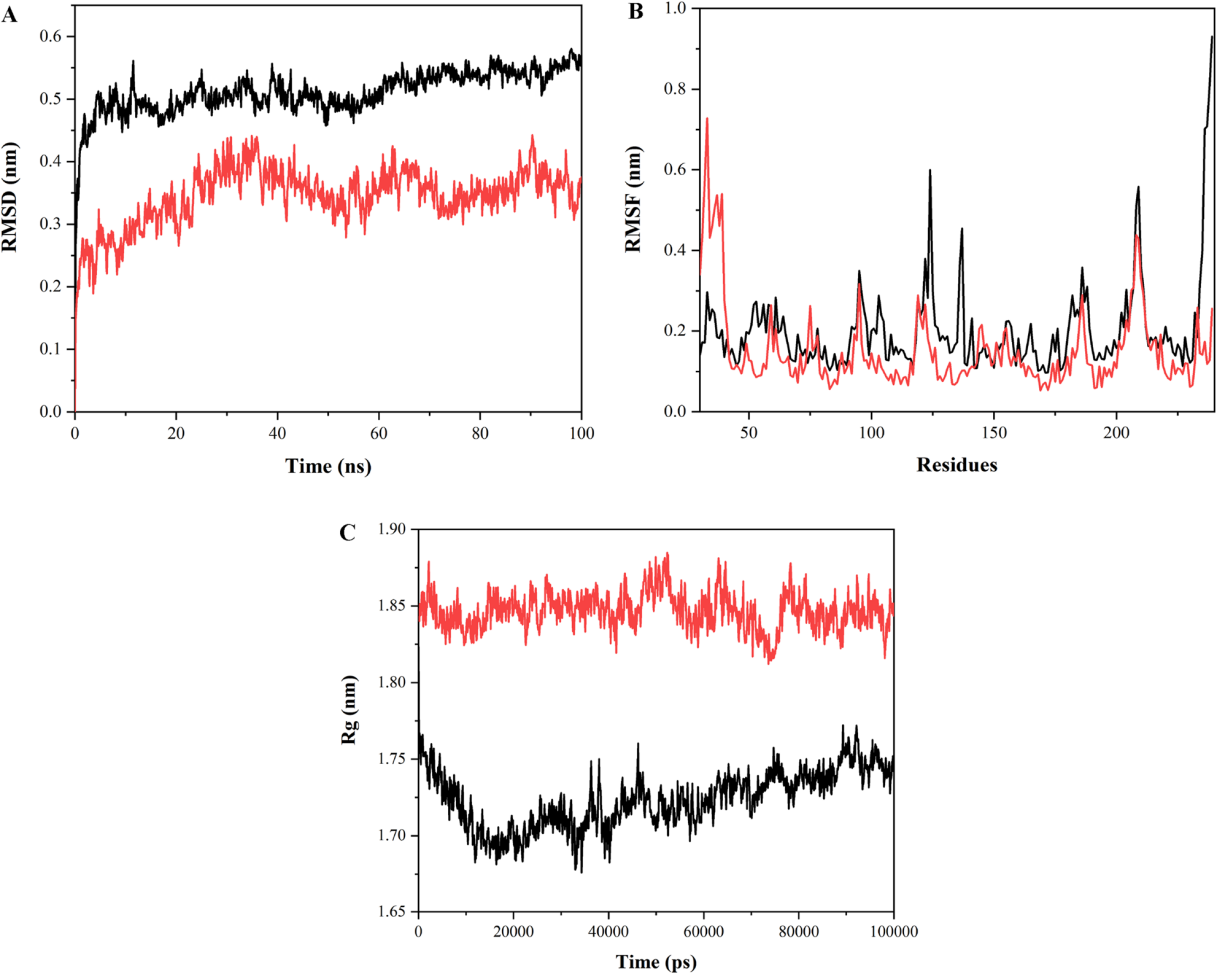
Molecular Dynamics Simulation data (**A**) RMSD plot of free protein (TasA) (shown in black), and their complex (TasA-4c) (shown in red) (**B**) RMSF values of free protein (TasA) (shown in black) and their complex (TasA-4c) (shown in red) (**C**) Rg values of free protein (TasA) (shown in black) and their complex (TasA-4c) (shown in red).

Root mean square deviation (RMSD) value obtained for the free protein (TasA) lies between 0.45 and 0.55 nm. The protein structure appears to be undergoing some changes in the structure. RMSD for the complex between the protein and ligand is seen to be fluctuating between 0.25 and 0.4 nm, lower than the RMSD for the free protein, and thus the complex formed appears to be stable (Fig. 8A). RMSF values for TasA are higher than the complex (TasA-4c) except for the initial few residues at the N-terminus where the complex exhibits fluctuations in RMSF values. The free protein shows a leap in RMSF for the residues at C-terminus (234–239 aa) (Fig. 8B). The radius of gyration (Rg) for the free protein lies between 1.68 and 1.75 nm, while for the complex, Rg ranges between 1.82 and 1.88 nm, which is mildly stretched throughout the simulation trajectory (Fig. 8C).

#### MD simulation of TapA-4c complex

**Figure 9:**
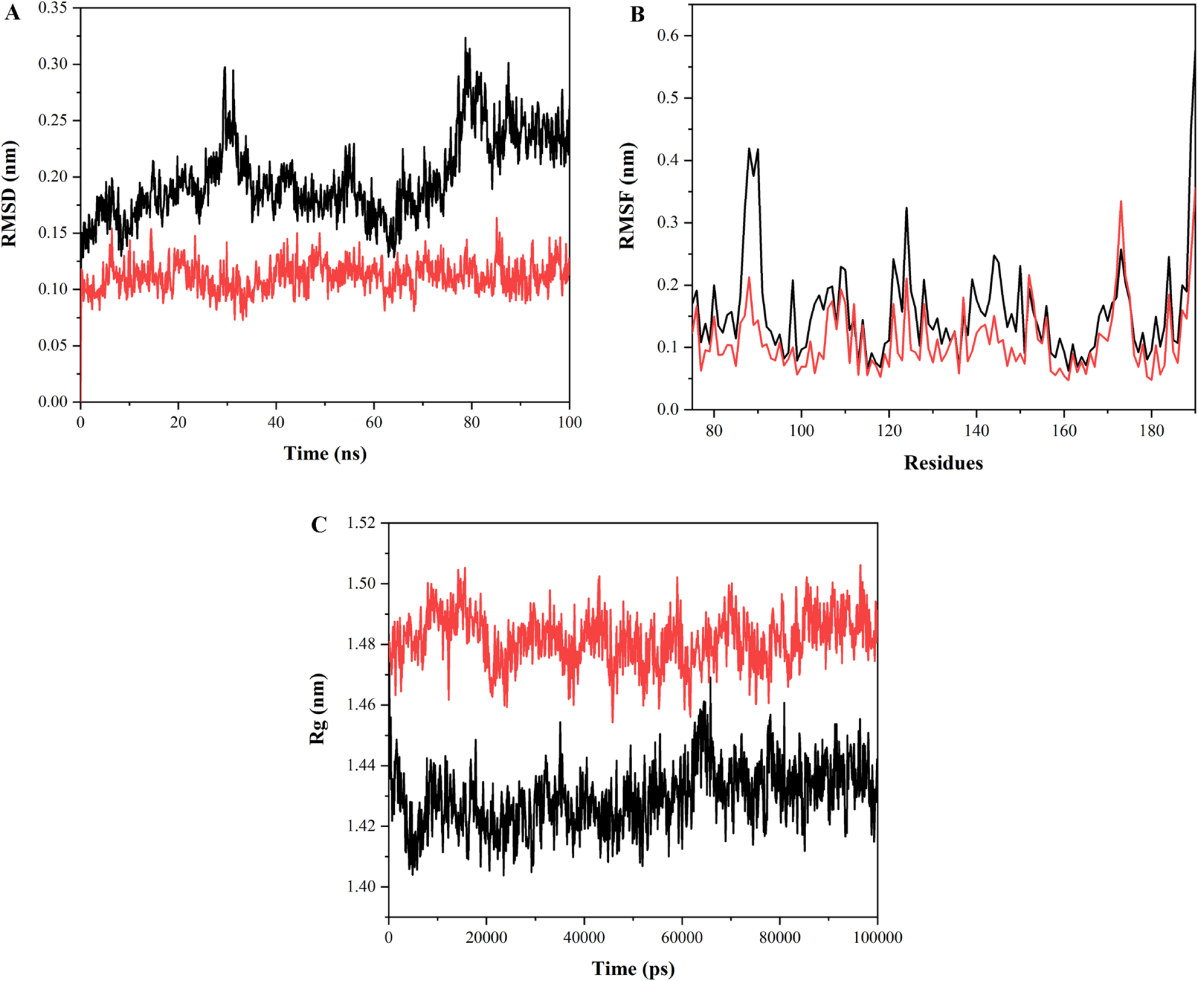
Molecular Dynamics Simulation data (**A**) RMSD plot of free protein (TapA) (shown in black), and their complex (TapA-4c) (shown in red) (**B**) RMSF values of free protein (TapA) (shown in black) and their complex (TapA-4c) (shown in red) (**C**) Rg values of free protein (TapA) (shown in black) and their complex (TapA-4c) (shown in red).

RMSD for TapA protein is between 0.13 and 3 nm including the fluctuations observed around 80 ns. 6HQC-4c complex exhibits RMSD between 0.07 and 0.15 nm, which is lower than RMSD of free protein and thus, the complex structure is observed to be highly stable and does not deviate from the initial structure. This further confirms stable complex formation between the protein and the ligand (Fig. 9A). RMSF for the free protein (TapA) ranges between 0.06 and 0.42 nm except for the leap up to 0.57 nm observed for the C-terminal residues. The complex RMSF is in the range of 0.05–0.35 nm throughout the trajectory (Fig. 9B). Rg values for the free protein are observed to be between 1.4 and 1.45 nm; similarly, Rg values for the TapA-4c complex are between 1.46 and 1.5 nm. The complex exhibits similar structural compactness as the free protein (Fig. 9C).

### ADMET analysis

Among all the compounds, only one (**4c**) violates one Lipinski’s Rule of Five due to molecular weight exceeding 500 Da. However, all these DHA compounds exhibit druglikeness. ADMET analysis for compounds **4a**–**e** is shown in Table 4. Water solubility is an indicator of solubility of the compound in water at 25 °C, and all these compounds are moderately soluble in water. Further, the permitted range for lipophilicity (LogP_o/w_ (iLOGP)) ranges from − 2 to 10, and all the compounds are well within the range. The analysis predicts that all the compounds possess a high intestinal absorption score. This indicates that these compounds would be better absorbed from the gastrointestinal tract when administered orally^75^. None of these compounds is able to permeate the blood–brain barrier (BBB). For a compound to qualify as a drug, the total number of H bond donors should not exceed 5. These include the bond formed between nitrogen (N) and hydrogen (H) and between oxygen (O) and hydrogen (H). For all the above-mentioned compounds, the H bond donors are less than 5. Similarly, the number of H bond acceptors should range from 0 to 10, and all the compounds conform to the limit. The synthesized compounds possess Topological Polar Surface Area (TPSA) ranging between 20 and 130 Å^2^, which is also within the acceptable range. It has been observed that for better CNS penetration, a compound should have lower TPSA values^76^.

**Table 4:** ADMET analysis for compounds 4a–e.

Properties	**4a**	**4b**	**4c**	**4d**	**4e**
Molecular weight (g/mol)	460.48	446.45	520.53	430.45	473.52
Water solubility	Moderate solubility				
Lipophilicity (Log P_o/w_) (iLOGP)	3.41	2.92	3.79	3.12	3.34
GI absorption	High				
BBB permeant	No	No	No	No	No
Lipinski rule (druglikeness)	Yes; 0 violation	Yes; 0 violation	Yes; 1 violation: MW > 500	Yes; 0 violation	Yes; 0 violation
Num. H-bond acceptors	7	7	9	6	6
Num. H-bond donors	2	3	2	2	2
Topological polar surface area (TPSA)	109.08 Å^2^	120.08 Å^2^	127.54 Å^2^	99.85 Å^2^	103.09 Å^2^

### Static biofilm assay

**Figure 10:**
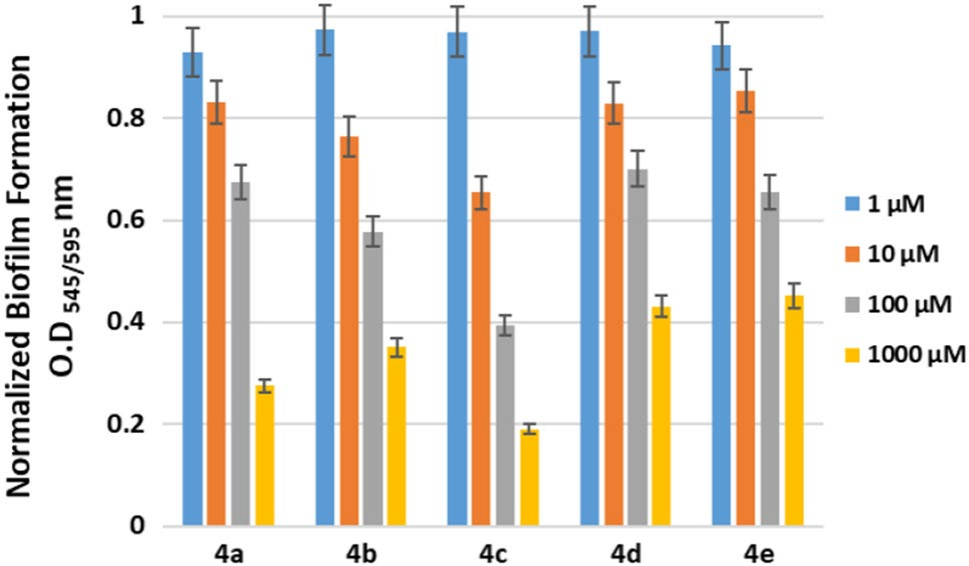
Static biofilm assay exhibiting inhibitory activity of DHA compounds (**4a**–**e**) against *Bacillus subtilis* biofilm.

The five synthesized DHA compounds (**4a**, **4b**, **4c**, **4d** and **4e**) were studied for their anti-biofilm activity, which was evaluated using static biofilm assay. The assay was performed to check the inhibitory activity of the compounds against biofilm formation by *B. subtilis* and *P. aeruginosa*. The results of the assay for antibiofilm activity against *B. subtilis* are shown in Fig. 10. As can be observed from the figure, all five synthetic substitutes show increased anti-biofilm activity with increasing concentrations of the compounds. Among these, DHA-pOMe (**4c**) showed the highest anti-biofilm activity with increasing concentration, followed by DHA-benz (**4a**), DHA-pOH (**4b**), DHA-pNMe2 (**4d**) and DHA-TriOMe (**4e**). The compound **4c** was found to significantly inhibit biofilm formation to the level of 19% at the highest concentration of 1000 µM (1 mM). Whereas the compounds **a**, **d**, **b**, and **e** showed inhibitory activity up to ~ 27%, 35%, 43% and ~ 45%, respectively at 1000 µM (1 mM). Overall, all the compounds were found to inhibit biofilm formation at a statistically significant percentage. The same trend was also observed for *P. aeruginosa* biofilm, i.e., **4c** (~ 26%), **4e** (~ 38%), **4a** (~ 39%), **4b** (~ 42%) and **4d** (~ 43%), and the data is shown in Supplementary Fig. 7.

### Minimum inhibitory concentration (MIC) assay

**Figure 11:**
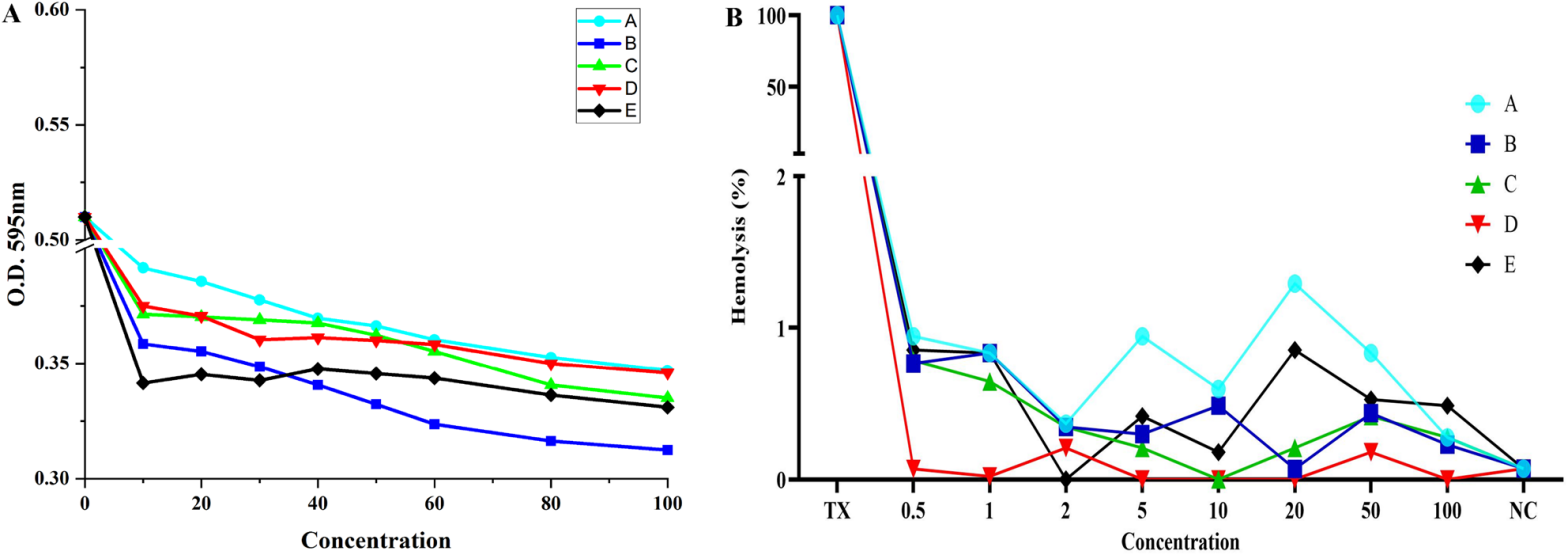
(**A**) MIC assay for compounds 4a–e against *Bacillus subtilis*. All the compounds lead to inhibition of bacterial growth in the range of 80–100 µM (**B**) Graphical representation of hemolytic activity of the test compounds 4a–e (0.5–100 µM) on treating with human erythrocytes. All the compounds exhibit low hemolysis (< 2%). Triton X-100 (TX) was used as positive control (100% hemolysis) and Erythrocytes in PBS was used as negative control (NC) (0% hemolysis). ****p < 0.0001 on comparison with positive control.

MIC of the test compounds were checked against *B. subtilis* as well as *P. aeruginosa*. For *B. subtilis*, all the compounds exhibit inhibition of bacterial growth in the range of 80–100 µM (Fig. 11A). For *P. aeruginosa* suspension, the compounds lead to growth inhibition in concentration between 80 and 100 µM. The figure showing MIC data for *P. aeruginosa* is given in Supplementary Information (Supplementary Fig. 8).

### Hemolytic assay

Hemolytic activity of the test compounds (**4a**–**e**) was checked at concentrations ranging from 0.5 to 100 µM on treatment with human erythrocytes as depicted in Fig. 11B. The detailed bar graphs of the data for individual compound are given in Supplementary Fig. 9. Even at the highest concentration tested (100 µM), all the compounds exhibited low hemolysis (< 2%). The data was analyzed statistically by using one-way analysis of variance (ANOVA) and was found to be significant (****p value < 0.0001). The data was analyzed using Graph Pad Prism software (Version 8.0.2, Graph Pad, San Diego, CA, USA). Low hemolysis is a good indicator for these compounds to be used for further in vivo studies.

### Cell viability assay

**Figure 12:**
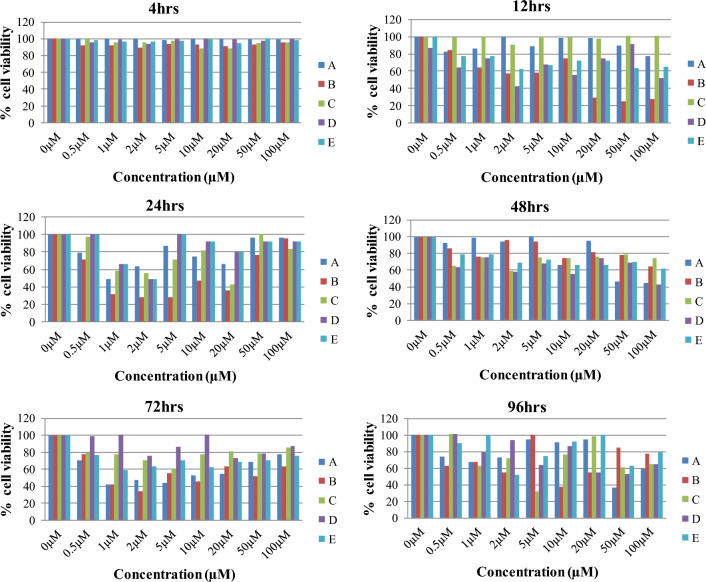
Bar graphs depicting percent viability of HEK-293 cells at various concentrations of compounds at different time points.

Figure 12 shows the percent cell viability of the human embryonic kidney cells (HEK-293) cells after treatment with compounds **4a**, **b**, **c**, **d** and **e** as a function of concentration. Similar treatment was performed for T24 cells and the data is shown in Supplementary Fig. 10. Notably, compounds **4a**–**e** exhibited no toxic effects on HEK-293 as well as T24 cells in concentrations ranging from 0.5 to 100 µM, whereas 0 µM was kept as blank (100% survival). Thus, it can be concluded that aforementioned compounds had no toxicity to cells and provide > 90% cell viability. Consequently, these compounds have a strong potential to be used as therapeutic agents without causing any toxicity to biological systems at the concentrations tested.

## Conclusion

This work reports the synthesis of novel dehydroacetic grafted spiro-oxindolo pyrrolidines involving [3 + 2]-cycloaddition reaction of azomethine ylides to various dehydroacetic acid derived chalcones derivatives via three-component reaction. The synthesized compounds possess diverse substitution patterns. This method is advantageous due to mild reaction conditions, operational simplicity, easy purification and selectivity with enhanced reaction rate in a one-pot three-component approach. Thus, the desired products were synthesized from the starting materials in a single step.

In silico studies provide crucial insights for studying the interaction of the molecules with a target protein. In this direction, molecular docking of the synthesized compounds was performed with biofilm forming *B. subtilis* proteins (TasA and TapA) and a *P. aeruginosa* protein (RetS kinase) (in Supplementary Information). All the compounds exhibited good binding with the target proteins, showing promising antibacterial activity. **4b** exhibits the best binding with TasA protein involved in biofilm formation. While the compound **4c** shows best binding with the TapA protein as per the docking studies, thus highlighting its potential anti-biofilm activity. This is also in concordance with the in vitro biofilm assay carried out in the current study. Out of all the synthesized compounds, **4c** showed good anti-biofilm activity against *B. subtilis* as well as *P. aeruginosa* biofilms in vitro. Molecular Dynamics simulations have been performed for the complex between TasA-4c and TapA-4c to confirm the formation of a stable protein–ligand complex. To check the antibacterial potential of the synthesized compounds, we have further performed MIC assay. Besides, potential of the compounds to cause hemolysis of human erythrocytes was checked by performing hemolytic assay. Interestingly, none of the compounds exhibited hemolysis even at the highest concentration tested. To determine the viability of cell lines on treatment with the compounds **4a**–**e**, cell viability assay was performed. The cells remained viable (> 90% viability) even till 96 h of treatment, which is an indicator of their non-toxicity. Further studies are needed to develop these compounds as novel antibacterial agents.

## Material and methods

DMEM, foetal bovine serum (FBS) and l-glutamine were purchased from Sigma-Aldrich, USA. Penicillin was purchased from HiMedia, India. HEK-293 and T24 cells were procured from NCCS, Pune, India. MTT reagent (3-(4,5-Dimethylthiazol-2-yl)-2,5-diphenyl tetrazolium bromide was purchased from HiMedia, India. DMSO was purchased from HiMedia, India.

All melting points are uncorrected. The Infra-Red (IR) spectra for all the compounds were recorded on an FT-IR instrument (JASCO 4700). ^1^H NMR spectra were recorded on a BRUKER 400 spectrometer at 300 MHz in CDCl_3_ or DMSO-d_6_ using TMS as the internal standard, while ^13^C NMR was recorded on the same spectrometer at 75 MHz. Mass spectra were recorded on LCMS 8045, SHIMADZU Scientific. Elemental analysis was carried out using FLASH 2000 HT Analyzer. UV–visible spectrum was recorded using Cary 5000 Model, NIR-UV Spectrometer. Silica gel (ACME, 100–200 mesh) was used for column chromatography. Reagent grade solvents were used as per the standard protocols. The starting materials such as dehydroacetic acid, sarcosine, isatin and hydrazine were purchased commercially and dehydroacetic acid-based chalcones were synthesized as described in the literature^46^.

### Synthesis of dehydroacetic acid grafted spiro-oxindolopyrrolidine heterocycles 4a–e

A mixture of dipolarophile **1a** (1 mmol), isatin **2** (1 mmol) and sarcosine **3** was refluxed in 20 mL of methanol until the reaction was completed, as evidenced by TLC in petroleum ether-ethyl acetate mixture (3.5:1.5). This was followed by visualization in an iodine chamber. After the reaction was completed, removal of the solvent was carried out at reduced pressure and the product (**4a**) was further purified by column chromatography with mixture of petroleum ether-ethyl acetate (4:1) as eluent.

### Spectroscopic data and physical properties for 4a–e

**1-N-Methyl-spiro-[2.3′]oxindole-3-[4″-hydroxy-6″-methyl-3″-carbonyl-2H-pyran-2″-one)-4-(*p*-methoxyphenyl)-pyrrolidine 4a** Color: pale yellow; Mp: 194–196 °C; IR (KBr): 3462, 3248, 1710, 1686, 1555, 1251 cm^−1^; ^1^H NMR (CDCl_3_/400 MHz): δ 2.05 (s, d, *J* = 0.4 Hz, 3H), 2.18 (s, 3H), 3.32 (dd, *J* = 7.4 Hz, 1.2 Hz, 1H), 3.67 (dd, *J* = 8.8 Hz, 2.0 Hz, 1H), 3.77 (s, 3H), 4.47–4.54 (m, 1H), 4.75 (d, *J* = 8.8 Hz, 1H), 5.71 (d, *J* = 0.8 Hz, 1H), 6.74 (d, *J* = 7.6 Hz, 1H), 6.68–6.88 (m, 3H), 6.98 (d, *J* = 7.2 Hz, 1H), 7.09–7.13 (m, 1H), 7.49–7.51 (m, 2H), 7.54 (s, 1H, NH); ^13^C NMR (CDCl_3_/100 MHz): δ 20.46, 34.44, 42.81, 55.28, 60.03, 64.37, 73.07, 100.32, 101.14, 109.45, 113.98, 122.17, 125.50, 126.90, 129.11, 129.70, 133.40, 142.22, 158.51, 160.77, 168.90, 179.45, 180.19, 204.24 ppm; LC–MS (Positive ionization mode): 461.25 [M + H]^+^. CHN analysis calculated for C_26_H_24_N_2_O_6_: C, 68.36; H, 5.25; N, 5.68; Found: C, 68.30.; H, 5.29; N, 5.41%.

**1-N-Methyl-spiro-[2.3′]oxindole-3-[4″-hydroxy-6″-methyl-3″-carbonyl-2H-pyran-2″-one)-4-(*p*-hydroxyphenyl)-pyrrolidine 4b** Color: pale yellow; Mp: 243–245 °C; IR (KBr): 3454, 1736, 1616, 1109, 472 cm^−1^; ^1^H NMR (DMSO-d_6_/400 MHz): δ 1.99 (s, 3H), 2.06 (s, 3H), 3.22 (t, *J* = 7.6 Hz, 1H), 3.44 (dd, *J* = 8.8 Hz, 1.6 Hz, 1H), 4.10 (bs, 1H), 4.29 (dd, *J* = 10.0 Hz, 8.2 Hz, 1H), 4.69 (d, *J* = 8.8 Hz, 1H), 6.07 (s, 1H), 6.68–6.71 (m, 2H), 6.76–6.82 (m, 2H), 6.88 (d, *J* = 8.4 Hz, 1H), 7.08–7.12 (m, 1H), 7.22 (d, *J* = 8.4 Hz, 1H), 7.62 (d, *J* = 8.4 Hz, 1H), 9.26 (s, 1H), 10.35 (s, 1H); ^13^C NMR (DMSO-d_6_/100 MHz): δ 20.33, 34.45, 42.38, 49.07, 59.74, 63.69, 72.97, 100.18, 101.25, 109.59, 115.71, 116.73, 121.50, 125.90, 127.02, 129.38, 129.54, 131.90, 156;55. 160.46, 169.98, 179.43, 183.25, 204.14 ppm; LC–MS (Positive ionization mode): 447.25 [M + H]^+^. CHN analysis calculated for C_25_H_22_N_2_O_6_: C, 66.38; H, 5.32; N, 5.84; Found: C, 66.16.; H, 4,54; N, 5.00%.

**1-N-Methyl-spiro-[2.3′]oxindole-3-[4″-hydroxy-6″-methyl-3″-carbonyl-2H-pyran-2″-one)-4-(3‴, 4‴, 5‴-trimethoxyphenyl)-pyrrolidine 4c** Color: pale yellow; Mp: 156–158 °C; IR (KBr): 3455, 1736, 1720, 1680, 1562, 1125 cm^−1^; ^1^H NMR (CDCl_3_/400 MHz): δ 2.06 (s, 3H), 2.18 (s, 3H), 3.38 (t, *J* = 6.6 Hz, 1H), 3.70 (t, *J* = 7.8 Hz, 1H), 3.81 (s, 3H), 3.90 (s, 6H), 4.47 (q, *J* = 6.8 Hz, 1H), 4.80 (d, *J* = 6.4 Hz, 1H), 5.73 (s, 1H), 6.76 (d, *J* = 6.0 Hz, 1H), 6.84–6.88 (m, 2H), 6.97 (d, *J* = 6.0 Hz, 1H), 7.11 (t, *J* = 6.2 Hz, 1H), 7.80 (s, 1H); ^13^C NMR (CDCl_3_/100 MHz): δ 20.47, 34.43, 56.15, 60.20, 60.80, 64.31, 73.24, 100.37, 101.15, 105.54, 109.55, 122.16, 125.39, 126.67, 129.18, 136.66, 137.55, 142.33, 153.19, 160.79, 169.02, 179.59, 180.17, 204.24 ppm. CHN analysis calculated for C_27_H_27_N_3_O_5_: C, 64.03; H, 5.42; N, 5.38; Found: C, 63.89.; H, 5.31; N, 5.30%.

**1-N-Methyl-spiro-[2.3′]oxindole-3-[6″-methyl-3″-carbonyl-2H-pyran-2″, 4″-(3H)-dione)-4-phenyl-pyrrolidine 4d** Color: pale yellow; Mp: 210–212 °C; IR (KBr): 3440, 1698, 1559, 1496, 754 cm^−1^; ^1^H NMR (CDCl_3_/400 MHz): δ 2.05 (d, *J* = 0.8 Hz, 3H), 2.19 (s, 3H), 3.36 (dd, *J* = 7.4 Hz, 1.2 Hz, 1H), 3.49 (s, 1H), 3.72 (dd, J = 8.8 Hz, 2.0 Hz, 1H), 4.52–4.59 (m, 1H), 4.81 (d, *J* = 9.2 Hz, 1H), 5.71 (d, *J* = 0.8 Hz, 1H), 6.76 (d, *J* = 7.6 Hz, 1H), 6.84–6.88 (m, 1H), 6.99 (t, *J* = 3.3 Hz, 1H), 7.09–7.14 (m, 1H), 7.19–7.23 (m, 1H), 7.29–7.33 (m, 2H), 7.57–7.59 (m, 2H), 7.66 (s, 1H, NH); ^13^C NMR (CDCl_3_/100 MHz): δ 20.46, 34.44, 43.54, 50.89, 59.94, 64.18, 73.09, 100.29, 101.15, 109.51, 122.17, 125.49, 126.81, 126.85, 128.58, 128.75, 128.98, 129.15, 141.37, 142.28, 160.79, 168.94, 179.46, 180.19, 204.10 ppm; LC–MS (Positive ionization mode): 431.70 [M + H]^+^. CHN analysis calculated for C_25_H_22_N_2_O_5_: C, 68.46; H, 5.43; N, 5.56; Found: C, 68.31.; H, 5.29; N, 5.41%.

**1-N-Methyl-spiro-[2.3′]oxindole-3-[6″-methyl-3″-carbonyl-2H-pyran-2″, 4″-(3H)-dione)-4-(*p*-*N*,*N*-dimethylaminophenyl)-pyrrolidine 4e** Color: pale yellow; Mp: 208–210 °C; IR (KBr): 3426, 1721, 1613, 1552, 1466 cm^−1^; ^1^H NMR (CDCl_3_/400 MHz): δ 2.04 (d, *J* = 0.4 Hz, 3H), 2.24 (d, *J* = 0.8 Hz, 3H), 2.90 (s, 6H), 3.06 (s, 1H), 3.30 (dd, *J* = 7.4 Hz, 1.2 Hz, 1H), 3.68 (dd, *J* = 8.8 Hz, 2.0 Hz, 1H), 4.44–4.51 (m, 1H), 4.78 (d, *J* = 9.2 Hz, 1H), 5.69 (d, *J* = 0.4 Hz, 1H), 6.70 (d, *J* = 8.4 Hz, 2H), 6.77 (d, *J* = 8.4 Hz, 2H), 6.83–6.87 (m, 1H), 6.99 (d, *J* = 7.2 Hz, 1H), 7.08–7.12 (m, 1H), 7.45 (d, *J* = 8.8 Hz, 2H), 8.04 (s, 1H); ^13^C NMR (CDCl_3_/100 MHz): δ 20.45, 34.50, 40.12, 40.77, 42.88, 60.07, 66.27, 73.12, 100.41, 101.14, 109.60, 111.77, 112.91, 122.04, 125.44, 127.03, 129.05, 129.16, 131.82, 142.49, 149.69, 160.81, 168.83, 179.69, 180.17, 204.54 ppm; LC–MS (Positive ionization mode): 474.25 [M + H]^+^. CHN analysis calculated for C_27_H_27_N_3_O_5_: C, 67.80; H, 5.56; N, 8.12; Found: C, 67.69.; H, 5.72; N, 8.25%.

### Molecular docking studies

#### Protein selection

Two proteins of the pathogen *B. subtilis* and one protein of *P. aeruginosa* were used as targets for the synthesized compounds. These proteins were selected in order to check antibacterial and, specifically, anti-biofilm activity of the synthesized compounds against *B. subtilis* and *P. aeruginosa* via molecular docking. The proteins were obtained from the RCSB PDB database and included TasA (PDB: 5OF1), TapA (PDB: 6HQC) and RetS kinase (PDB: 6DK8).

The biofilm matrix formed by *B. subtilis* consists of two components—TasA, a protein producing amyloid-fibers and an exopolysaccharide (EPS). The entire matrix gets assembled with the help of another protein BslA. The resulting biofilm is known to be hydrophobic in nature, and this property is attributed to BslA as well as EPS components essential for its formation^77^. TapA protein is essential for TasA fiber assembly. Strains expressing mutant protein lacking 8 residues positioned at the N terminal region led to defects in biofilm formation and even delay in the formation of biofilm in vivo^78^. Chronic *P. aeruginosa* infection is the result of biofilm formation in lungs of the host. During formation of biofilm, various effectors of acute infection are downregulated while the production of exopolysaccharides is enhanced. The shift between acute and chronic modes of infection is regulated by GacS/GacA system. GacS further interacts with and is regulated by signaling kinases RetS and LadS, indicating the importance of RetS for the formation of biofilm by the pathogen^79^.

#### Ligand selection

The synthesized compounds **4a**–**4e** have been used as ligands for molecular docking studies with *B. subtilis* proteins TasA (PDB: 5OF1), TapA (PDB: 6HQC) and *P. aeruginosa* protein RetS kinase (PDB: 6DK8).

#### Molecular docking and analysis

Molecular docking is a widely used approach for the prediction of protein–ligand interactions. Blind docking was performed to decipher interactions among the synthesized ligands with the selected proteins. Pymol is used to visualize the protein files used for the study as well as for the results generated. OpenBabel (http://openbabel.org/wiki/Category:Installation) was used for converting all the ligand files originally in ChemDraw (.cdx) format to PDBQT format. Finally, AutoDock Vina was used for performing docking. A similar approach was used for all the ligands. The final analysis is done using binding energies and inhibition constants thus obtained after docking is completed. After successful completion of docking, the protein–ligand complex is further analyzed for detecting residues involved in hydrophobic and H-bond interactions by protein–ligand interaction profiler (PLIP) (https://plip-tool.biotec.tu-dresden.de/plip-web/plip/index)^80^. LigPlot + was used for the visualization of hydrogen bonding and hydrophobic interactions^81^.

### Molecular Dynamics (MD) Simulations

Molecular Dynamics simulations were performed to understand the conformational dynamics of the complex using GROMACS tool through the webserver WebGro from Sim Lab (https://simlab.uams.edu/)^82^. MD simulations were performed for the protein, ligand as well as the protein–ligand complexes exhibiting good binding affinity in concordance with the biofilm assay. MD simulations of TasA protein (PDB: 5OF1) and TasA-4c complex, and TapA protein (PDB: 6HQC) and TapA-4c complex were performed at 100 ns. GROMOS96 43a1 forcefield was used for simulations. The triclinic box was filled with Simple Point Charge (SPC) water molecules. Na^+^ and Cl^−^ ions were added in order to neutralize the system. The Steepest Descent integrator was selected for energy minimization. Berendsen NVT ensemble was used for simulating the system and also equilibrated using NPT. Simulations were performed for trajectory of 100 ns at 300 K with a pressure of 1 bar.

### ADMET analysis

Absorption, distribution, metabolism, excretion and toxicity (ADMET) provides information on the pharmacokinetic properties of a compound of interest. An ideal drug candidate besides being effective against the target molecule should also possess non-toxicity at therapeutic doses so that the side-effects upon administration are minimal. Various pharmacodynamic activities of a compound like carcinogenicity, brain penetration, bioavailability etc. can be determined using this analysis. The SwissADME server has been used for the current work (http://www.swissadme.ch/)^83^. The Lipinski’s Rule of Five includes molecular weight (default range 50–500 Da), iLOGP (octanol/water partition coefficient, ranging from − 2 to 10), HBAs (H bond acceptors, 0–10), and HBDs (H bond donors, 0–5)^84^. As per Rules of Five, a compound cannot not be orally active if there is a violation of two or more than two rules^85^. For instance, poor absorption reflects that the metabolism well as the distribution of the drug would be hampered, which may ultimately lead to nephrotoxicity and even neurotoxicity^86^. The Topological Polar Surface Area (TPSA) of a compound provides information about surfaces belonging to polar atoms. The higher the TPSA, the lesser is the permeability across the membrane^75^.

### Static biofilm assay

Bacterial cultures (*P. aeruginosa* and *B. subtilis*) were grown overnight and Optical Density (O.D.) at 595 nm (~ 1.5) was adjusted to McFarland standard (10^8^ cfu/mL).This was followed by dilution to 20 fold (1:20) using sterile nutrient broth to finally obtain 10^5^ cfu/mL with appropriate volumes (1–1000 µM) of compounds **4a**–**e**. 200 µL of this suspension was then aliquoted into a 96-well microtiter plate (TPP®; polystyrene). This was followed by incubation at 37 °C for 24 h without agitation. Each compound was evaluated in triplicate. For this assay, OD of the suspended bacterial culture was measured after 24 h without any treatment at 595 nm using a microplate reader (TECAN, Synergy, USA). The suspended cells were discarded and the plate was washed with 1 × phosphate-buffered saline (PBS) to remove the suspended cells. The sessile biofilm cells were then stained with 1.0% crystal violet (1 g crystal violet in 20% methanol) for 30 min. Following this, the plate was washed with autoclaved Milli-Q water to remove the remaining unbound dye. The stained biofilm cells were eluted by pure ethanol and further quantified by checking OD at 545 nm. The biofilm formation was quantified as the OD at 545 nm (amount of crystal violet bound to biofilms) divided by O.D. at 595 nm (the amount of suspended cells)^87^.

### Minimum inhibitory concentration (MIC) assay

The Minimum inhibitory concentration (MIC) assay was performed to check the minimum concentration of the compounds (**4a**–**e**) under investigation that kill 50% of the bacterial growth. MIC was determined using the microdilution method as per the Clinical and Laboratories Standard Institute (CLSI) guidelines. Different concentrations of the test compounds were prepared (10–100 µM). *B. subtilis* and *P. aeruginosa* suspensions was diluted to 1 × 10^8^ cfu/mL, in order to obtain McFarland scale turbidity (0.5), with O.D. in the range of 0.08–0.1 at 625 nm. Following this, the bacterial culture was diluted 1:200 in Mueller Hinton broth (MHB) to obtain 5 × 10^5^ cfu/mL. This bacterial suspension was then added to the 96-well microtiter plate, along with the test compounds in 1:1 ratio. Sterile MHB and bacterial suspension served as negative and positive controls, respectively. At the end of 24 h incubation at 37 °C, MIC was determined spectroscopically using microplate reader at 595 nm^88^. The assays were performed in triplicates.

### Hemolytic assay

For evaluating the potential of the synthesized compounds (**4a**–**e**) to release hemoglobin in the plasma, in vitro hemolysis assay was performed. The erythrocytes were harvested from healthy human blood by centrifugation in sterile PBS (pH = 7.4) at 2000 rpm for 10 min. Erythrocytes were washed with PBS at least thrice, followed by preparation of 2% solution (v/v) in fresh PBS. 100 μL of this solution was added to 100  μL of compound concentrations (ranging from 0.5 to 100 μM). The suspension was further incubated for 1 h at 37 °C. 2% (v/v) Triton X-100 was used as positive control for complete (100%) hemolysis, while PBS was used as the negative control (NC) for no hemolysis. After incubation, the suspension was centrifuged at 2200 rpm for 5 min, followed by addition of the supernatant to the 96-well microtiter plate. The O.D. was measured at 540 nm to detect the amount of released hemoglobin as a measure of red blood cell lysis^89^. Percentage of hemolysis was then calculated by the following equation:

$$ \begin{aligned} {\text{Percentage}}\;\left( \% \right){\text{ hemolysis}} & = {\text{O}}{\text{.D}}{.}\;{\text{of}}\;{\text{test}}\;{\text{concentration}} - {\text{O}}{\text{.D}}{.}\;{\text{of}}\;{\text{Negative}}\;{\text{control}}/{\text{O}}{\text{.D}}{.}\;{\text{of }} \\ & \quad {\text{Positive}}\;{\text{control}} - {\text{O}}{\text{.D}}{.}\;{\text{of}}\;{\text{Negative}}\;{\text{control}} \\ \end{aligned} $$

The blood was obtained from healthy human control at PGIMER, Chandigarh. All methods were approved and carried out in accordance with relevant guidelines and regulations of the PGIMER institutional ethics committee (Ethical clearance number PGI/IEC/2022/000734). Informed consent was obtained from the healthy donor.

### Cell viability assay

Cytotoxicity of compound A, B, C, D and E were performed using an MTT assay. HEK-293 and T24 cells were seeded in a 96-well microtiter plate at cell density of 2000 cells/well in growth medium consisting of DMEM with 5% fetal bovine serum and antibiotics (100U/mL penicillin and 3 mM glutamine). Cells were incubated for 24 h at 37 °C and 5% CO_2_/95% air humidified atmosphere. Subsequently, at different time points viz 4 h, 12 h, 24 h, 48 h and 96 h, different concentrations (0.5–100 µM) of compounds **4a**, **b**, **c**, **d** and **e** were incubated into the growth medium, after which 100  μL of MTT dye was added (at the respective time points) to each well, and the plate was further incubated for 4 h. This was followed by addition of 100 µL of solubility reagent (Dimethyl sulfoxide, DMSO) to each well. The quantity of formazan color was measured spectrophotometrically at 570 nm (ELISA reader, Bio-Rad). Survival percentage was calculated in comparison to blank cells (100% survival). The cell viability was determined according to the following equation:

$$ \% {\text{ Cell}}\;{\text{Proliferation}}\left( {\% {\text{ living cells}}} \right) = \left( {{\text{Mean}}\;{\text{O}}{\text{.D}}{.}\;{\text{Sample}} - {\text{Mean}}\;{\text{O}}{\text{.D}}{.}\;{\text{Negative}}\;{\text{Control}}} \right)*100 $$

### Ethical approval

The blood was obtained from healthy human control at PGIMER, Chandigarh. All methods were approved and carried out in accordance with relevant guidelines and regulations of the PGIMER institutional ethics committee (Ethical clearance number PGI/IEC/2022/000734). Informed consent was obtained from the healthy donor.

## Supplementary Information

Supplementary Information.

## Data Availability

The data underlying this study are available in the published article and its Supporting Information.

## Abbreviations

*B.** subtilis*: *Bacillus subtilis*
CK-MB: Creatine kinase MB isoenzyme
DHA: Dehydroacetic acid
EPS: Exopolysaccharide
MCR: Multicomponent reaction
MM2: Molecular mechanics
*P.** aeruginosa*: *Pseudomonas aeruginosa*
