# Author Correction: Evaluation of antibiofilm properties of dehydroacetic acid (DHA) grafted spiro-oxindolopyrrolidines synthesized via multicomponent 1,3-dipolar cycloaddition reaction

**DOI:** 10.1038/s41598-025-90897-1

**Published:** 2025-03-06

**Authors:** Adukamparai R. Suresh Babu, Akanksha Sharma, M. P. Athira, Hema K. Alajangi, A. R. Naresh Raj, Janeka Gartia, Gurpal Singh, Ravi Pratap Barnwal

**Affiliations:** 1 Department of Chemistry, Anna University, Chennai, 600025 India; 2 Department of Biophysics, Panjab University, Chandigarh, 160014 India; 3 University Institute of Pharmaceutical Sciences, Panjab University, Chandigarh, 160014 India; 4 Department of Chemistry, IISER, Mohali, Sahibzada Ajit Singh Nagar, Punjab, 140306 India; 5 Department of Chemistry, Dwaraka Doss Goverdhan Doss Vaishnav College, Arumbakkam, Chennai, 600106 India; 6 School of Biotechnology, Kalinga Institute of Industrial Technology (KIIT), Bhubaneswar, Odisha 751024 India; 7 Department of Chemistry, University of Calicut, Thenhipalam, 673635 India

Correction to: *Scientific Reports* 10.1038/s41598-023-42528-w, published online 15 September 2023

The original version of this Article omitted an affiliation for Adukamparai R. Suresh Babu and M. P. Athira. The correct affiliations are listed below.

Adukamparai R. Suresh Babu

Department of Chemistry, Anna University, Chennai, 600025, India

Department of Chemistry, University of Calicut, Thenhipalam 673635, India

M. P. Athira

Department of Chemistry, IISER, Mohali, Sahibzada Ajit Singh Nagar, Punjab, 140306, India

Department of Chemistry, University of Calicut, Thenhipalam 673635, India

The original Article has been corrected.

