# Supplementary material for: Evaluation of antibiofilm properties of dehydroacetic acid (DHA) grafted spiro-oxindolopyrrolidines synthesized via multicomponent 1,3-dipolar cycloaddition reaction
Source: Sci Rep. 2023 Sep 15;13:15289. doi: 10.1038/s41598-023-42528-w (PMC10504327; doi:10.1038/s41598-023-42528-w)
Supplement: Supplementary file 1 — Supplementary Information. [file 41598_2023_42528_MOESM1_ESM.docx]

**1-N-Methyl-spiro-[2.3’]oxindole-3-[4’’-hydroxy-6’’-methyl-3’’-carbonyl-2H-pyran-2’’-**

**one)-4-(*p*-methoxyphenyl)-pyrrolidine 4a**

**1-N-Methyl-spiro-[2.3’]oxindole-3-[4’’-hydroxy-6’’-methyl-3’’-carbonyl-2H-pyran-2’’-one)-4-(*p*-hydroxyphenyl)-pyrrolidine 4b**

**1-N-Methyl-spiro-[2.3’]oxindole-3-[4’’-hydroxy-6’’-methyl-3’’-carbonyl-2H-pyran-2’’-one)-4-(3’’’, 4’’’, 5’’’-trimethoxyphenyl)-pyrrolidine 4c**

**1-N-Methyl-spiro-[2.3’]oxindole-3-[6’’-methyl-3’’-carbonyl-2H-pyran-2’’, 4’’-(3H)-dione)-4-phenyl-pyrrolidine 4d**

**1-N-Methyl-spiro-[2.3’]oxindole-3-[6’’-methyl-3’’-carbonyl-2H-pyran-2’’, 4’’-(3H)-dione)-4-(*p*-*N*,*N*-dimethylaminophenyl)-pyrrolidine 4e**

**Compounds Details**

**Supplementary Figure 1.** Interaction of TasA (PDB: 5OF1) with **A.** 4a **B.** 4b **C.** 4c. **D.** 4d and **E.** 4e. The ligands are shown in black color

**Supplementary Figure 2:** 2D interaction figures for interaction of TasA with **A.** 4a **B.** 4b **C.** 4c **D.** 4d and **E.** 4e. Figures have been generated using LigPlot^+^ [1]. Red dotted lines represent hydrophobic interactions and green dotted lines depict hydrogen bonds.

**Supplementary Figure 3.** Interaction of TapA (PDB: 6HQC) with **A.** 4a **B.** 4b **C.** 4c. **D.** 4d and **E.** 4e. The ligands are shown in black color

**Supplementary Figure 4:** 2D interaction figures for interaction of TapA with **A.** 4a **B.** 4b **C.** 4c **D.** 4d and **E.** 4e. Figures have been generated using LigPlot^+^ [1]. Red dotted lines represent hydrophobic interactions and green dotted lines depict hydrogen bonds.

**Molecular dockings and analysis of *Pseudomonas aeruginosa* protein RetS kinase with synthesized compounds**

Docking of the RetK kinase protein with all the ligands (4a-e) were carried out using AutoDock Vina. Among all the ligands, 4e showed lowest binding energy of -7.5 kcal/mol, followed by 4b and 4d (-7.4 kcal/mol each). The inhibition constants were also calculated and are provided in supplementary table 1. Amino acids involved in various interactions with the ligands have also been summarized. Interestingly, GLU462 and THR465 are involved in hydrophobic interactions with all the ligands except 4a. THR465 is also involved in hydrogen bonding with all the ligands except 4a along with LYS544. The interactions of all these ligands with RetS kinase are depicted in figures prepared using PyMol and shown as supplementary figure 4. Further, and hydrogen bonding and hydrophobic interactions of the protein with the ligands are shown in supplementary figure 5.

**Supplementary table 1:** Binding energy, inhibition constant and various residues of the protein RetS kinase involved in hydrogen bonding and hydrophobic interactions with ligands 4a-e

| **Compound** | **Protein (RetS kinase (PDB: 6DK8))** | | | |
| --- | --- | --- | --- | --- |
|  | **Binding energy (kcal/mol)** | **Inhibition constant (µM)** | **Hydrophobic interactions** | **H-bonding** |
| 4a | -5.9 | 46.81 | PHE418 | ALA416, PHE418, THR465 |
| 4b | -7.4 | 3.71 | PHE418, GLU462, THR465 | THR465, LYS544 |
| 4c | -7 | 7.29 | PHE418, GLU462, THR465 | THR465, LYS544 |
| 4d | -7.4 | 3.71 | GLU462, THR465, LEU466 | THR465, LYS544 |
| 4e | -7.5 | 3.13 | GLU462, THR465, LEU466 | THR465, LYS544 |

**Supplementary Figure 5:** Interaction of RetS kinase (PDB: 6DK8) with **A.** 4a **B.** 4b **C.** 4c. **D.** 4d and **E.** 4e. The ligands are shown in black color.

**Supplementary figure 6:** 2D interaction figures for interaction of RetS kinase with **A.** 4a **B.** 4b **C.** 4c **D.** 4d and **E.** 4e. Figures have been generated using LigPlot^+^. Red dotted lines represent hydrophobic interactions and green dotted lines depict hydrogen bonds.

**Supplementary figure 7:** A static biofilm assay showing inhibitory activity of DHA compounds (4a-e) against *Pseudomonas aeruginosa* biofilm.

**Supplementary Figure 8:** MIC assay for compounds 4a-e against *Pseudomonas aeruginosa*. All the compounds lead to inhibition of bacterial growth in the range of 80-100 µM

**Supplementary Figure 9: A.** Results of hemolytic assay for the test compounds **B.** Positive control (Erythrocytes in 2% v/v Triton X-100) showing 100% hemolysis of erythrocytes **C.** Negative control (Erythrocytes in PBS) showing no hemolysis **D.** Bar graph representation of hemolytic activity of the test compounds 4a-e (0.5-100 µM) on treating with human erythrocytes. All the compounds exhibit low hemolysis (<2%). Triton X-100 (TX) was used as positive control (100% hemolysis) and Erythrocytes in PBS was used as negative control (NC) (0% hemolysis). ****p<0.0001 on comparison with positive control

**Supplementary Figure 10:** Bar graphs depicting percent viability of T24 cells at various concentrations of compounds at different time points

**Supplementary Figure 11: FT-IR spectrum of the product 4a**

**Supplementary Figure 12: ^1^H NMR spectrum of 4a**

**Supplementary Figure 13: ^1^H NMR spectrum of 4a (Expanded)**

**Supplementary Figure 14: ^13^C NMR spectrum of 4a**

**Supplementary Figure 15: Mass spectrum of 4a**

**
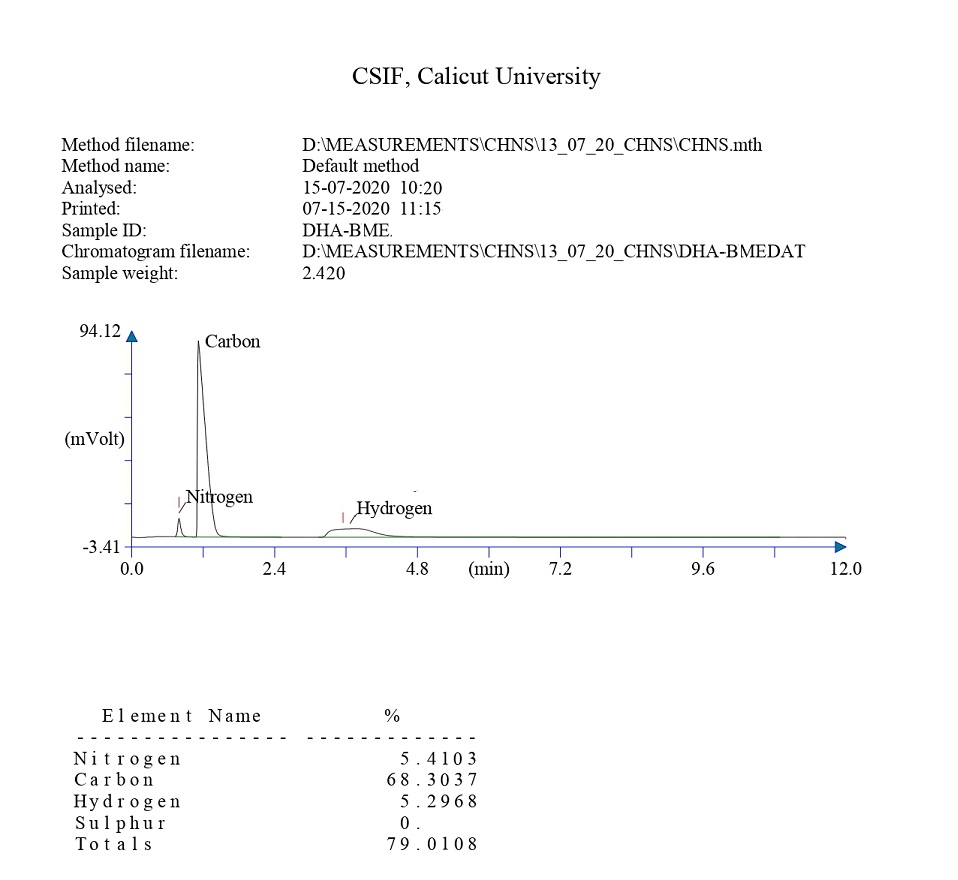
**

**Supplementary Figure 16: Elemental analysis of the product 4a**

**Supplementary Figure 17: FT-IR spectrum of 4b**

**Supplementary Figure 18: ^1^H NMR spectrum of 4b**

**Supplementary Figure 19: ^1^H NMR spectrum of 4b (Expanded)**

**Supplementary Figure 20: ^13^C NMR spectrum of 4b**

**Supplementary Figure 21: Mass spectrum of 4b**

**
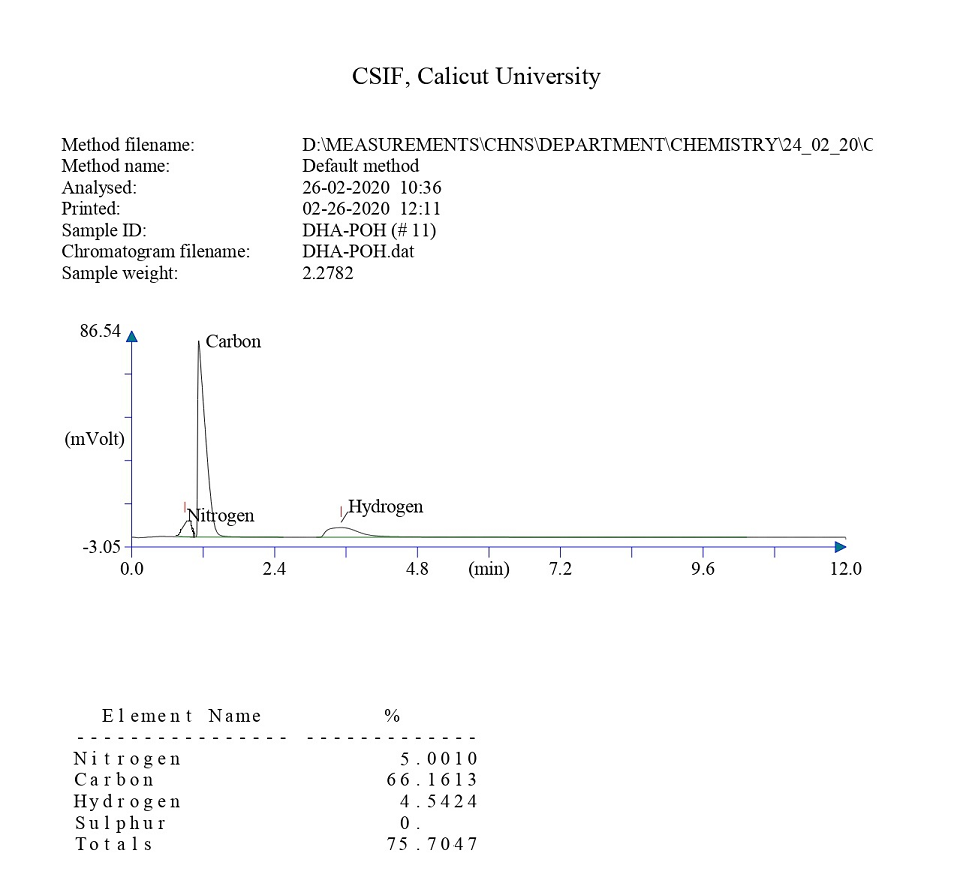
**

**Supplementary Figure 22: Elemental analysis of the product 4b**

**Supplementary Figure 23: FT-IR spectrum of 4c**

**Supplementary Figure 24: ^1^H NMR spectrum of 4c**

**Supplementary Figure 25: ^1^H NMR spectrum of 4c (Expanded)**

**Supplementary Figure 26: ^13^ C NMR spectrum of 4c**

**
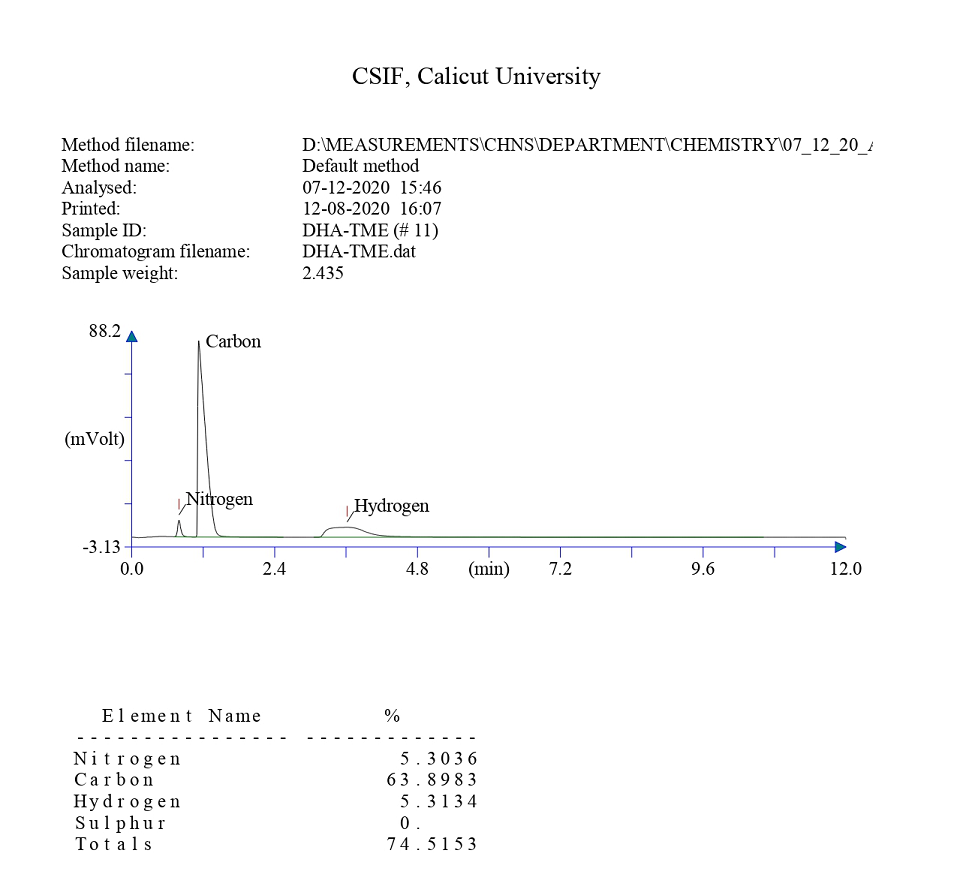
**

**Supplementary Figure 27: Elemental analysis of the product 4c**

**Supplementary Figure 28: FT-IR spectrum of 4d**

**Supplementary Figure 29: ^1^H NMR spectrum of 4d**

**Supplementary Figure 30: ^13^C NMR spectrum of 4d**

**Supplementary Figure 31: Mass spectrum of 4d**

**
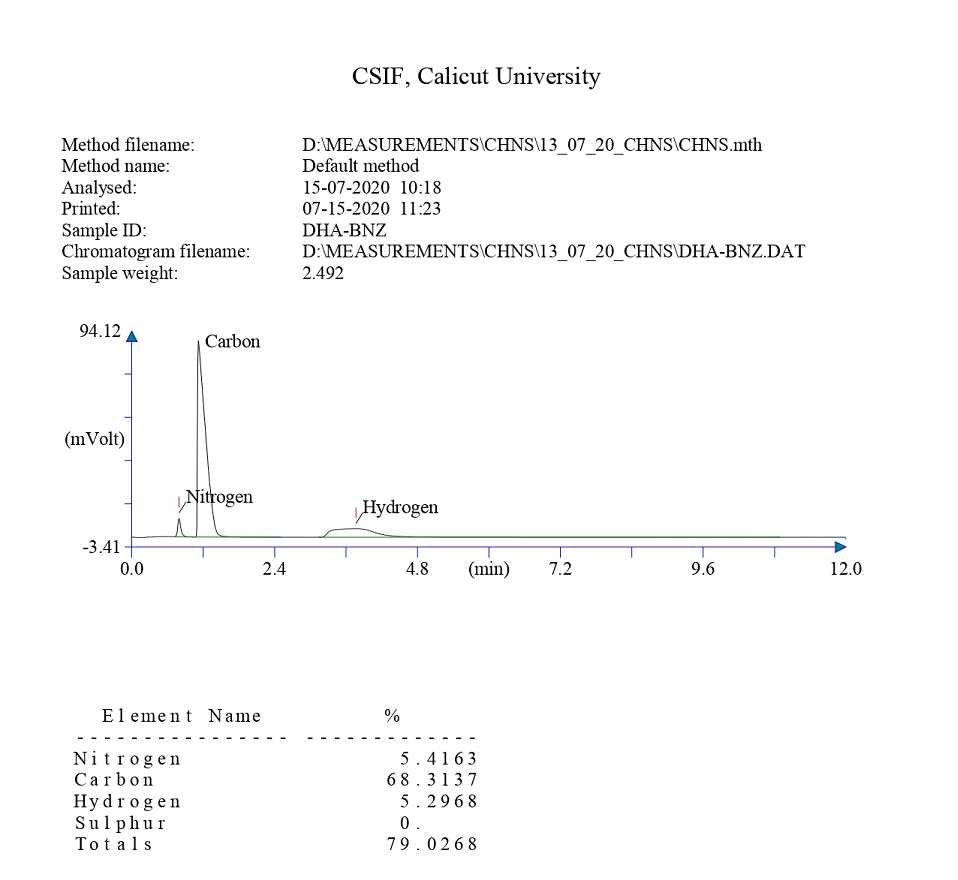
**

**Supplementary Figure 32: Elemental analysis of the product 4d**

**Supplementary Figure 33: FT-IR spectrum of 4e**

**Supplementary Figure 34: ^1^H-NMR spectrum of 4e**

**Supplementary Figure 35: ^1^H NMR spectrum of 4e (Expanded)**

**Supplementary Figure 36: ^13^C NMR spectrum of 4e**

**Supplementary Figure 37: Mass spectrum of 4e**

**
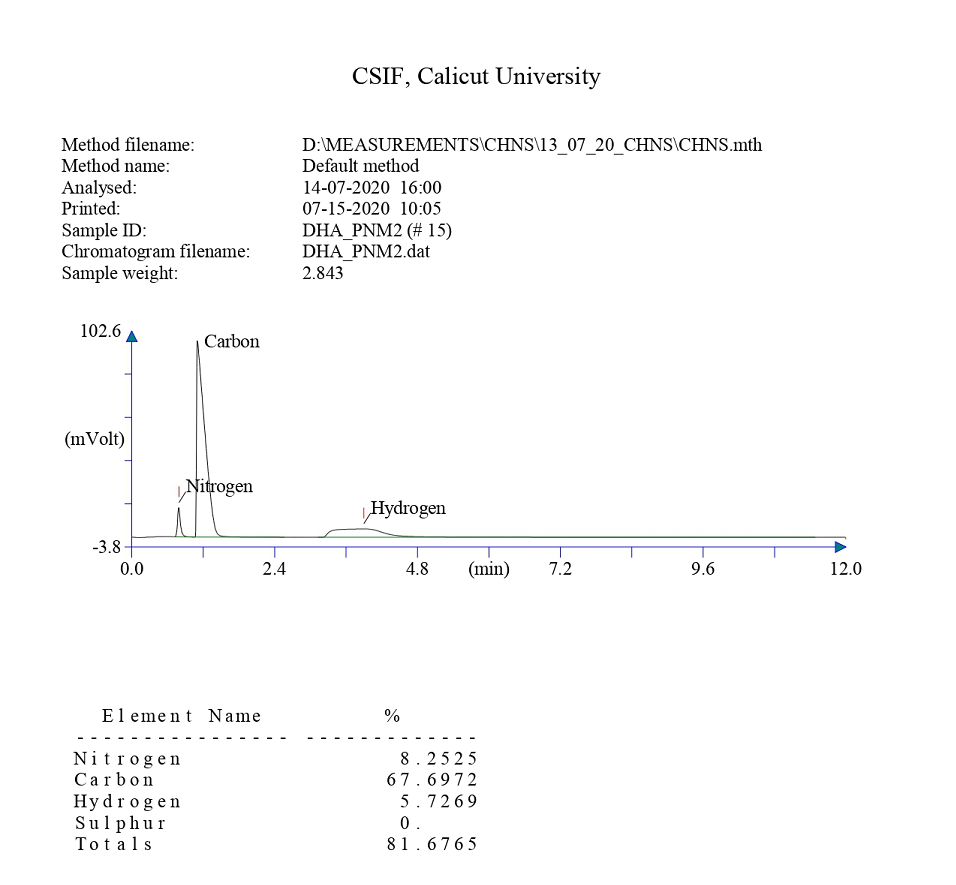
**

**Supplementary Figure 38: Elemental analysis of the product 4e**

**References:**

[1] R.A. Laskowski, M.B. Swindells, LigPlot+: Multiple Ligand–Protein Interaction Diagrams for Drug Discovery, Journal of Chemical Information and Modeling 51(10) (2011) 2778-2786.
